# Effective treatment of non-fusion RET intragenic deletion lung adenocarcinoma with pralsetinib: a case report

**DOI:** 10.3389/fmed.2025.1546287

**Published:** 2025-06-18

**Authors:** Wenjun Li, Wenjun Zhu, Can Yang, Yaping Tian, Linna Cao, Lirong He

**Affiliations:** ^1^Department of Respiratory and Critical Care Medicine, The Second Affiliated Hospital of Nanchang University, Nanchang, Jiangxi, China; ^2^Department of Respiratory and Sleep Medicine, Peking University People’s Hospital, Beijing, China

**Keywords:** intragenic RET deletion, lung adenocarcinoma, pralsetinib, next-generation sequencing, targeted therapy

## Abstract

RET fusions, the most common oncogenic RET alterations, occur in approximately 1–2% of non-small cell lung cancer (NSCLC) cases and represent well-established therapeutic targets. Pralsetinib, a selective RET kinase inhibitor, has demonstrated significant efficacy and tolerability in patients with RET fusion-positive NSCLC. However, the clinical management of NSCLC with non-fusion RET structural variants remains challenging. Here, we report a case of a middle-aged male diagnosed with stage IV lung adenocarcinoma, in whom initial next-generation sequencing (NGS) revealed no actionable mutations. The patient achieved a partial response to pemetrexed and platinum-based chemotherapy, but disease progression occurred 9 months later. Upon re-biopsy, a large intragenic RET deletion involving exons 2–11 was detected. Based on this finding, the patient was treated with pralsetinib and achieved radiological tumor regression, with a progression-free survival of 5 months to date. This case highlights a potential therapeutic role for RET inhibitors even in the absence of canonical fusions, and underscores the importance of reassessing the tumor’s molecular profile following treatment failure, as acquired genomic alterations may provide new targets for precision therapy.

## Introduction

Lung cancer remains the leading cause of cancer-related mortality worldwide (1, 2), with adenocarcinoma being the most common histologic subtype of non-small cell lung cancer (NSCLC), accounting for approximately 40% of all cases (3). Among the various molecular drivers identified in NSCLC, Rearranged during transfection (RET) gene fusions are relatively rare but well-established oncogenic alterations, present in approximately in 1–2% of cases (4). Located on chromosome 10q11.21, the RET gene encodes a single-pass transmembrane receptor tyrosine kinase. RET gene fusions arise from chromosomal rearrangements that juxtapose the RET kinase domain with the dimerization domain of a partner gene, leading to ligand-independent activation of oncogenic signaling pathways (5). RET gene fusions are more frequently found in non-smokers, younger patients, and those with non-squamous histology, and are associated with a higher incidence of brain metastases and a low-immune-infiltration tumor microenvironment (6). Pralsetinib, a highly selective RET tyrosine kinase inhibitor, has demonstrated significant antitumor activity and good tolerability in patients with RET fusion-positive metastatic NSCLC (7), and has been approved for this indication (8). However, clinical evidence regarding the efficacy of pralsetinib in patients with non-fusion RET structural variants, such as intragenic deletions, remains limited. Here, we report the case of a middle-aged male patient with stage IV lung adenocarcinoma harboring a large RET intragenic deletion involving exons 2–11, without detectable RET gene fusion. The patient received pralsetinib as second-line therapy and achieved a notable clinical response.

## Case presentation

**Figure 1:**
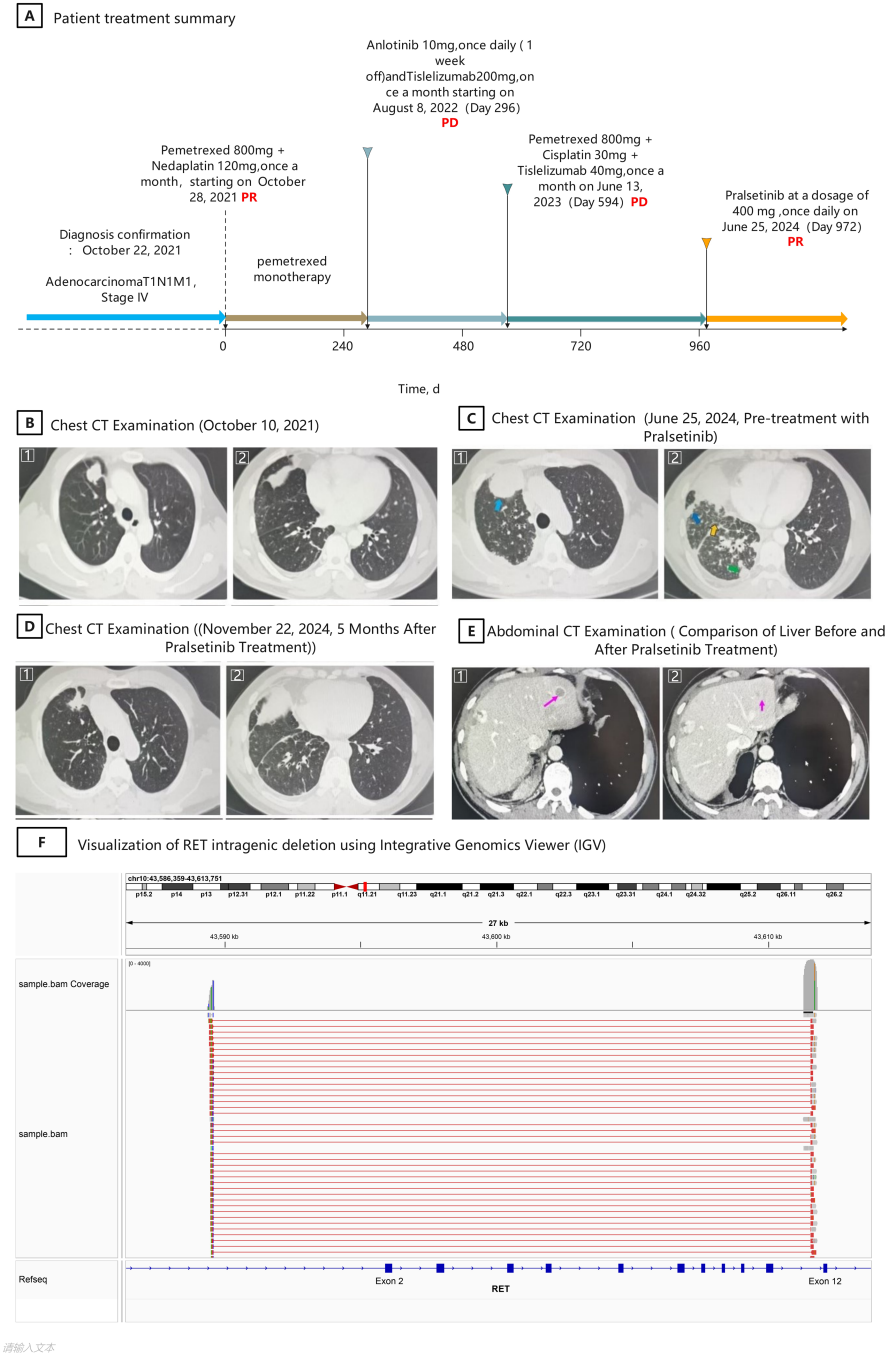
**(A)** Schematic summary of treatment timeline. **(B)** Chest CT on October 10, 2021: **(B1)** Right upper lobe pulmonary nodule measuring 29 × 20 mm. **(B2)** Right pleural thickening with post-drainage changes of pleural effusion. **(C)** Chest CT on June 25, 2024 (prior to pralsetinib initiation): **(C1)** Right upper lobe mass enlarged to 54 × 39 mm. **(C2)** Arrows (left to right) indicate: pleural septal thickening, carcinomatous lymphangitis, and pleural nodularity. **(D)** Chest CT on November 22, 2024 (after 5 months of pralsetinib treatment): **(D1)** Tumor shrinkage in the right upper lobe to 19 × 22 mm. **(D2)** Resolution of carcinomatous lymphangitis and pleural lesions. **(E)** Abdominal CT comparison before and after pralsetinib treatment: **(E1)** June 25, 2024: multiple metastatic liver nodules observed. **(E2)** November 22, 2024: complete resolution of liver metastases. **(F)** Visualization of RET intragenic deletion using Integrative Genomics Viewer (IGV). IGV screenshot showing next-generation sequencing data aligned to the RET gene on chromosome 10. Coverage plots and read alignment reveal a large intragenic deletion spanning exons 2 to 11. Reads are aligned to regions flanking the deletion (exons 1–2 and exon 12), with absence of coverage in the intervening exons. Split-read alignments further support a direct junction between exon 2 and exon 12. The deletion results in loss of the extracellular domain (including cadherin-like and cysteine-rich regions), while preserving the intracellular tyrosine kinase domain.

A 49-year-old male with a history of heavy smoking presented with chest tightness and dyspnea (Figure 1A). On October 22, 2021, computed tomography (CT) revealed nodules in the right upper lung lobe, substantial pleural effusion, and atelectasis of the right lower lobe (Figure 1B). Brain magnetic resonance imaging (MRI), contrast-enhanced abdominal CT, and whole-body bone scan revealed multiple nodules in the right lung, with evidence of metastatic disease involving the right chest, abdomen, and diaphragm, along with irregular masses. A CT-guided lung biopsy confirmed lung adenocarcinoma. DNA and RNA extracted from the initial biopsy were analyzed using a targeted 26-gene NGS panel based on semiconductor sequencing technology (Novogene, China). This panel was designed primarily to detect canonical point mutations and gene fusions in common lung cancer drivers. RET fusions were interrogated at the RNA level, but the panel lacked coverage for non-fusion structural alterations, such as large intragenic deletions. Consequently, no actionable alterations were reported at the time. This underscores an important limitation of many targeted panels and highlights the potential for false-negative results when evaluating only canonical fusion events. Cytological examination of the pleural effusion confirmed adenocarcinoma of pulmonary origin (TTF1 positive). PD-L1 testing was performed using the Dako 22C3 antibody, and the combined positive score (CPS) was approximately 1; TPS and IC scores were not reported. According to the 8th edition of the American Joint Committee on Cancer (AJCC) staging system (9), the patient was diagnosed with stage IV lung adenocarcinoma (T1N1M1), with an Eastern Cooperative Oncology Group (ECOG) performance status of 3.

On October 28, 2021, the patient began 4 cycles of chemotherapy with pemetrexed and nedaplatin, achieving a partial response and significant improvement in symptoms. This was followed by maintenance therapy with pemetrexed monotherapy, during which the patient remained progression-free for 9 months, with an improved ECOG performance status of 1. However, on July 13, 2022, a follow-up chest CT revealed an enlarged mass in the right upper lobe and a new pleura metastasis. The ECOG performance status had declined to 2. As a second-line treatment, the patient was switched to anlotinib (10 mg orally, once daily, 2 weeks on, 1 week off) in combination with tislelizumab, starting on August 8, 2022. Unfortunately, a follow-up CT scan on May 22, 2023 showed disease progression, with increased pleural effusion and further enlargement of the right upper lobe mass. A repeat CT-guided biopsy was recommended, but the patient initially declined.

On June 13, 2023, treatment with tislelizumab, pemetrexed, and cisplatin was initiated. After 4 cycles, disease progression persisted. Upon obtaining informed consent, a re-biopsy was performed. NGS of the second biopsy was conducted using the Burning Rock® Dx platform, which applies DNA-based hybrid capture and high-throughput sequencing technology. This comprehensive panel covers the full exonic regions of nine key NSCLC driver genes and the intronic hotspots of three additional genes. It is capable of detecting single nucleotide variants, copy number changes, and structural variants, including gene fusions. The assay identified a large intragenic deletion involving exons 2–11 of the RET gene, with a variant allele frequency (VAF) of 9.63% (Figure 1F). IGV visualization of the sequencing data demonstrated an abrupt loss of read coverage from exons 2 through 11, while exons 12 to 20 remained intact. Split-read alignments supported a direct breakpoint between exon 2 and exon 12, consistent with a structural deletion that eliminates the extracellular domain but preserves the intracellular tyrosine kinase domain. According to the molecular diagnostics report, this alteration was classified as a non-fusion structural variant of uncertain significance (VUS), and no fusion partner gene was detected. The deletion retained the RET kinase domain, suggesting a possible role in aberrant RET signaling. PD-L1 expression was not reassessed in the second biopsy due to limited tissue and the prioritization of molecular profiling. Additionally, the patient had previously demonstrated only low PD-L1 expression (CPS ≈ 1), and had already received immunotherapy in the second-line setting without benefit. At that time, no RET-targeted therapy was yet approved or widely available in mainland China for either RET fusion-positive or non-fusion RET-altered NSCLC. The patient initially declined further treatment. Although the RET exon 2–11 deletion was classified as a VUS, it was the only detectable alteration in the progressive tumor, and it involved the functional kinase domain of RET, suggesting potential oncogenic activity. In the context of prior treatment failure, absence of other actionable mutations, and the plausible functional impact of this non-canonical structural variant, a clinical decision was made to initiate trial use of pralsetinib (400 mg orally once daily), which had previously demonstrated efficacy and good tolerability in RET fusion-positive NSCLC (7, 10). The patient began pralsetinib on June 25, 2024 (Figure 1C). According to the Response Evaluation Criteria in Solid Tumors (RECIST) (11), the patient achieved a partial response, with a progression-free survival (PFS) of 5 months as of November 2024 (Figure 1D). Follow-up CT imaging also showed resolution of liver metastases compared to pre-treatment scans (Figure 1E). No treatment-related adverse events were observed during the course of pralsetinib therapy.

## Discussion

This case highlights the therapeutic potential of pralsetinib in a patient with lung adenocarcinoma harboring a non-fusion intragenic RET deletion, representing a rare and understudied molecular subset of NSCLC. While RET fusions are well-established oncogenic drivers occurring in approximately 1–2% of lung adenocarcinomas, other RET alterations—including point mutations, amplifications, and structural deletions—are much less common, and their oncogenicity and therapeutic relevance remain incompletely characterized (12). Consequently, it is currently unclear whether clinical guidelines developed for RET fusion–positive NSCLC are applicable to non-fusion RET-altered tumors, making this case particularly relevant.

In this patient, NGS identified a large intragenic deletion involving RET exons 2–11, eliminating the extracellular cadherin-like repeats, the cysteine-rich domain (CRD), and the transmembrane region, while preserving the intracellular tyrosine kinase domain. This CRD, stabilized by disulfide bonds including those involving Cys634, maintains RET in an autoinhibited conformation by preventing dimerization. Cryo-EM studies have shown that CRD forms a C-clamp structure that masks the hydrophobic transmembrane region, limiting spontaneous activation (13). Functionally, RET constructs lacking the extracellular domain exhibit increased autophosphorylation at key residues such as Y1062, indicating enhanced kinase accessibility (14). Moreover, the RET kinase domain can adopt an active-like conformation even in the absence of phosphorylation, due to a lack of strong cis-autoinhibitory elements (15). These findings support the hypothesis that structural disruptions like exon 2–11 deletions can drive constitutive RET activation, providing a rationale for RET-targeted therapy.

Although the RET exon 2–11 deletion was initially classified as a variant of uncertain significance (VUS), it was the only acquired alteration detected at progression. In the absence of other actionable mutations, pralsetinib was initiated, leading to partial radiologic response and 5 months of progression-free survival (PFS), without significant toxicity, suggesting RET pathway dependency. While most clinical data on pralsetinib derive from RET fusion–positive NSCLC (10), its efficacy in non-fusion RET alterations is less well established. Furthermore, recent clinical observations suggest that selective RET inhibitors may have activity in other non-fusion RET alterations such as amplifications and point mutations. For instance, Gandhi et al. reported a case of lung adenocarcinoma harboring high-level wild-type RET amplification (22–28 copies), without other known oncogenic alterations, that responded to selpercatinib with confirmed systemic and intracranial responses. This represents the first documented clinical benefit of selective RET inhibition in a patient with RET-amplified, non-fusion NSCLC (16). These early findings underscore the therapeutic relevance of RET pathway dependence beyond fusion events and support broader exploration of selective RET inhibitors in molecularly selected populations. Although data remain limited, the response observed in this RET-amplified case highlights the need for further prospective studies to evaluate the efficacy of RET inhibitors in non-fusion RET-altered NSCLC.

This case also highlights the importance of repeated molecular profiling. The RET deletion was not detected at diagnosis but emerged at progression, illustrating the dynamic nature of tumor evolution and underscoring the need for serial genomic testing to uncover newly actionable events (17).

## Conclusion

Pralsetinib may represent a viable therapeutic option for patients with non-fusion RET intragenic deletions, as evidenced by the favorable response observed in the presented case. Structural plausibility, observed clinical benefit, and the absence of alternative drivers together support the potential oncogenicity and druggability of this alteration. However, further studies are warranted to better characterize clinical outcomes and define optimal treatment strategies for this rare subset of NSCLC. Clinicians should remain vigilant for the emergence of acquired alterations during the disease course and consider repeat molecular profiling at progression, as such reassessment may uncover previously undetected actionable targets.

## Data Availability

The datasets presented in this study can be found in online repositories. The names of the repository/repositories and accession number(s) can be found in the article/supplementary material.
